# Bad Air Can Also Kill: Residential Indoor Air Quality and Pollutant Exposure Risk during the COVID-19 Crisis

**DOI:** 10.3390/ijerph17197183

**Published:** 2020-09-30

**Authors:** Samuel Domínguez-Amarillo, Jesica Fernández-Agüera, Sonia Cesteros-García, Roberto Alonso González-Lezcano

**Affiliations:** 1 Instituto Universitario de Arquitectura y Ciencias de la Construcción, Escuela Técnica Superior de Arquitectura, Universidad de Sevilla, 41014 Sevilla, Spain; sdomin@us.es; 2 Escuela Politécnica Superior, Universidad San Pablo-CEU, Montepríncipe Campus, Boadilla del Monte, 28668 Madrid, Spain; son.cesteros.ce@ceindo.ceu.es (S.C.-G.); rgonzalezcano@ceu.es (R.A.G.-L.)

**Keywords:** indoor air quality, COVID 19, European, dwellings, buildings, TVOC, PM_2.5_, CO_2_ concentration

## Abstract

During the first outbreak of the SARS-CoV-2 pandemic the population, focusing primarily on the risk of infection, was generally inattentive to the quality of indoor air. Spain, and the city of Madrid in particular, were among the world’s coronavirus hotspots. The country’s entire population was subject to a 24/7 lockdown for 45 days. This paper describes a comparative longitudinal survey of air quality in four types of housing in the city of Madrid before and during lockdown. The paper analysed indoor temperatures and variations in CO_2_, 2.5 μm particulate matter (PM_2.5_) and total volatile organic compound (TVOC) concentrations before and during lockdown. The mean daily outdoor PM_2.5_ concentration declined from 11.04 µg/m^3^ before to 7.10 µg/m^3^ during lockdown. Before lockdown the NO_2_ concentration values scored as ‘very good’ 46% of the time, compared to 90.9% during that period. Although the city’s outdoor air quality improved, during lockdown the population’s exposure to indoor pollutants was generally more acute and prolonged. Due primarily to concern over domestic energy savings, the lack of suitable ventilation and more intensive use of cleaning products and disinfectants during the covid-19 crisis, indoor pollutant levels were typically higher than compatible with healthy environments. Mean daily PM_2.5_ concentration rose by approximately 12% and mean TVOC concentration by 37% to 559%. The paper also puts forward a series of recommendations to improve indoor domestic environments in future pandemics and spells out urgent action to be taken around indoor air quality (IAQ) in the event of total or partial quarantining to protect residents from respiratory ailments and concomitantly enhanced susceptibility to SARS-CoV-2, as identified by international medical research.

## 1. Introduction

**Figure 1:**
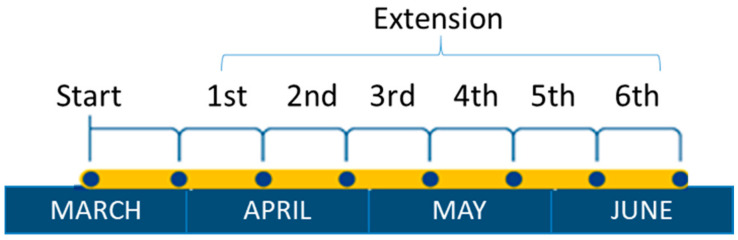
Chronology and periods of the state of alarm in Spain in 2020.

On 11 March 2020, the World Health Organization elevated the categorization of the public health emergency caused by COVID-19 to an international pandemic [1]. Since the COVID-19 pandemic began [2], after the first positive case detected in Spain on 30 January 2020, the number of cases detected increased exponentially until the Spanish government approved a Royal Decree 463/2020 on 14 March, declaring a state of emergency for the management of the health crisis situation caused by COVID-19 [3], forcing the closure of all non-essential economic activities, schools, and sports activities, as well as a ban on travel in Spain. During the state of alarm, people were only allowed to travel on public roads to complete basic activities, with the obligation to stay at home for the rest of the time. Figure 1 and Table 1 show the chronology of the different phases of the state of alarm in Spain.

Given this situation, millions of Spanish citizens remained confined to their homes until the beginning of the de-escalation phases. This confinement meant that the population, which previously did not spend more than 60% of their daily time in the home, were now spending 100% of their time in the home on a continual basis.

The city of Madrid was one of the countries hardest hit by the coronavirus, both in terms of the number of people infected per million inhabitants and the total number of deaths. The mortality rate in Spain was 54.73 deaths per 100,000 inhabitants; in Madrid it was 312 deaths per 100,000 inhabitants on 5 May 2020 [4,5]. 

**Figure 2:**
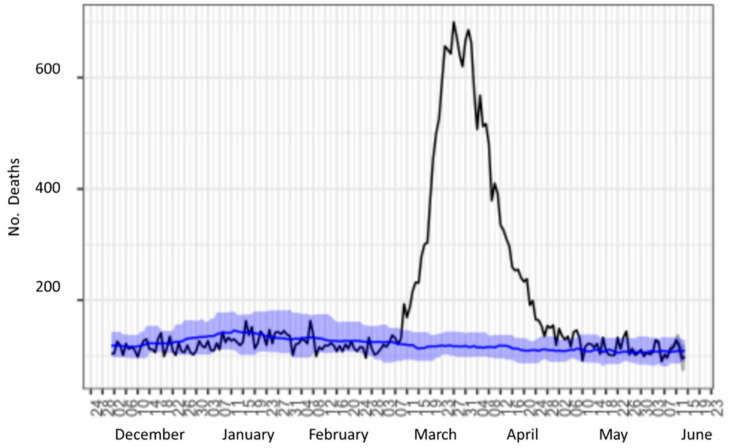
Mortality from all expected and observed causes in the community of Madrid (blue shading represents the average evolution of the previous 10 years).

Figure 2 shows the mortality from all expected and observed causes in the community of Madrid (blue shading represents the average evolution of the previous 10 years). Various studies on the emotional impact of the coronavirus episode in the first days of confinement in Spain [6,7] explored the relationship between a comprehensive set of lifestyles, sociodemographic variables, and personality and mood variables during the early stages of quarantine. The presence of low tolerance to negative emotions, neuroticism, and, to a lesser extent, friendliness, sleep quality, the presence of children, and time spent surfing the Internet presented the strongest correlations with the population’s moods. A similar study was conducted in Portugal, in which the lifestyle habits, anxiety levels, and basic psychological needs (BPN) in Portuguese adults during the COVID-19 pandemic were characterized, including a comparison between sex and age groups [8]. Other studied parameters of interest include the population’s activity level and anxiety [9], and the relationship with addiction [10].

The measures implemented by the governments of different countries have caused numerous environmental impacts, some of which can be considered positive, such as the improvement in air and water quality in urban areas, whereas others have been negative, such as the deterioration of the quality of coastal effluents due to the elimination of sanitary supplies [11]. The improvement in air quality has been particularly notable in large cities in China, where outdoor pollution levels (NO_2_ and PM_2.5_) have decreased significantly during lockdown [12,13]. In European cities like Barcelona, NO_2_ concentrations were reduced by half during the lockdown, PM decreased lower, and O_3_ concentrations increased by around 50% [14]. PM_2.5_ deterioration of air quality may lead to various kinds of pulmonary and cardiovascular diseases causing premature death [15]. In addition, there are several studies that indicate cities with poorer outdoor air quality have had a higher death rate in those affected by COVID-19 [16,17,18,19,20].

The reduction in environmental pollution in cities is a positive aspect for their inhabitants, which can contribute to improving living conditions during confinement by allowing for healthier ventilation. During this period, most of the population has been exclusively confined to their homes, so they were exposed to the same indoor atmosphere almost 100% of the time. In many cases, these homes are not entirely suitable for intensive use, with little interior space in relation to the number of inhabitants and, on many occasions, without adequate systems for thermal comfort, ventilation, and indoor air renewal [21,22,23].

Although the most common type of housing in Europe is single-family, this situation is different in Southern Europe, where collective residential apartment buildings predominate [24], especially in social housing [25] and in the larger proportion of social housing in the current general housing stock [26,27]. Although with regional variations, the architecture and construction of multi-family buildings are similar throughout the area [28], differing from the usual solutions in central and northern European countries. Many of these architectural solutions are a legacy of the residential construction boom of the 1960s and 1970s [26,28]. These southern European inhabitants also share cultural and social similarities both in the way people live in their homes and in managing their ventilation, heating and cooling system [21,29,30].

Household users are exposed to several pollutants. There are numerous source of pollution produced by the occupants and their activities, by the furniture and building materials, by technical defects, the presence of humidity or dirt, and, in particular, the use of chemicals (cleaning, hygiene, and personal care products) as well as tobacco smoke in smokers’ homes [31,32,33,34]. Pollution from outside such as pollen, dust, and industrial and traffic pollution must also be considered. Through a review of the literature, Lozano and Siegel provided evidence for most of the countries studied of the prevalence of poor environmental quality conditions along with a lack of thermal comfort in social housing units [35].

There are numerous studies on indoor quality and sick building syndromes, especially in cold countries [36,37,38,39,40,41], but studies in temperate areas of Southern Europe are limited, with some studies in operational housing conditions [42,43,44,45,46,47]. Studies of the indoor air quality in homes during the COVID 19 lockdown are also limited, although some studies point to household cleaning products as a particularly relevant source of indoor pollution. Many people may be cleaned more frequently and with stronger disinfectants to reduce the potential of viral infection. Efforts to make homes airtight to improve energy efficiency have created buildings with reduced outdoor ventilation rates resulting in the buildup of indoor pollutants to harmful levels that would be otherwise unacceptable outdoors [48].

The aim of this study was to determine the impact of COVID-19-related confinement on the indoor air quality of dwellings in Madrid. The concentrations of CO_2_, PM_2.5,_ and TVOCs were measured over time before and during lockdown in relation to the thermal-physical parameters of indoor air quality to identify health risks of the occupants due to this continuous exposure and the alteration of the usual cycles in the dwellings.

## 2. Materials and Methods

### 2.1. Sampling

Madrid is the capital of Spain. The population of the city is about 3,182,981, although if the floating population and the metropolitan area are considered, this number exceeds 6,685,471 inhabitants [49]. Located at 40°41′ and an altitude of 659 m above sea level, its climate is characterized by a transition between the cold semi-arid (Köppen BSk) and the Mediterranean (Csa), with an average annual temperature of 15 °C. Winters are moderately cold, with average temperatures in the coldest month (January) of around 6 °C. Summers are hot, with the average temperature exceeding 25 °C in July, with average maximum temperatures of between 32 and 33.5 °C.

The daily temperature oscillation is significant in the city periphery (around ∆t 13 K), but is less so in the city urban area (daily ∆t goes usually below 10 K). The annual thermal amplitude is also high: between 19 and 20 °C. This climatic context traditionally favored the use as naturally-ventilated houses promoting a semi-open state with most windows open for a significant portion of the year, a trend that is declining in the southern European cities due to outdoor pollution and other factors, such as noise and behavioral pattern changes. This situation makes the area especially relevant as a typical situation for analyzing the behavior of the household stock, which shared many building and behavioral characteristics with other southern European urban agglomerations during the pandemic. The most widespread residential buildings are multi-family dwellings, either in linear or H-shaped blocks [50].

**Figure 3:**
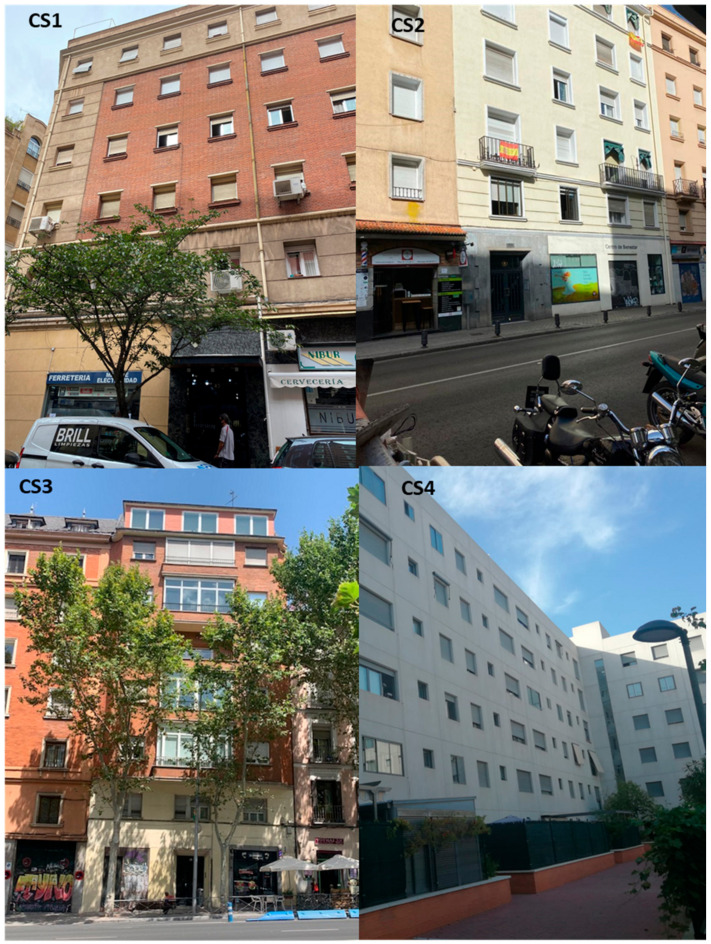
Case study typologies.

For this work, four case studies representative of the typical legacy social housing in the city, were selected for monitoring their environmental conditions under actual operational conditions before and during the state of confinement. Figure 3 shows the case study typologies. The typical construction of these buildings—mainly brick masonry and reinforced concrete—results in very compartmentalized housing units, with the apartments as unitary volume units. Generally, the buildings lack common elements that allow air communication between units (there are typically no chimneys, or common ventilation ducts). Previously conducted assessments [51] showed that the air leakage ratios to adjacent units did not exceed 4% of the air flow (tests at 50 Pa pressurization). For practical purposes, it is possible to consider the apartment units as independent from each other.

The selected case studies represent the typical legacy social housing in the city plus one contemporary development. Different profiles of occupants with enough representativeness of the structure of the use of national dwellings were selected. The case studies were selected due to the characteristics of the building and the family composition, to provide a sample of the most common types of families: from a single adult living alone (CS3), which represents 25.3% of the population of Spain between the elderly and under 65s living alone, a couple with one child (21%), a couple with two children (17.6%), and a couple with two children and a dependent elderly person (5.7% of Spain’s total but growing quickly).

The example selection was developed from the INFILES National R&D Project (on residential buildings infiltration national assessment) [52,53], due to the representativeness of national housing (the prototypical characteristics, both in morphology and construction and equipment). The daily hours of occupation refer to the average occupation of each one of the groups of inhabitants before the stay-at-home order—business as usual occupation—and when the lockdown period was in force (figures in brackets) when the inhabitants mostly remained at home (apart from essential workers, the population could only leave home to make quick purchases of basic items) almost continuously until the beginning of the deconfinement.

### 2.2. Monitoring

The monitoring period ran from 1 February to 30 April 2020. Indoor data (thermal and pollutants) were recorded at an interval of 15 min using electronic sensors and a data logger system. Detailed information on the characteristics of the measurement equipment is in Appendix A. Thermal ambient monitoring was conducted in accordance with ISO7726:1998 specifications (ISO/TC 159/SC 5, 1998), both in terms of the instrumentation and methods [42]. The system was in continuous operation during both time periods. The outdoor environmental data were provided by the Air Quality Service of Madrid Council (Station 28,079,007 of the Air Quality network). The outdoor air quality station was particularly representative both for its location, barycentric to the cases in the urban center and for the reliability of the measurements. The case studies were located around the station with distances ranging from 2 to 4.33 km from it, all of which are located within the consolidated urban area of the city (not in the suburbs). The variations with respect to other stations in the area were not very significant in general. The general climate data were provided by the Spanish national meteorological office (AEMET) [54]. Portable reference sensors were used to record periodic contrast measurements in situ to ensure the consistency and accuracy of the measurements of the monitoring systems.

### 2.3. User Information Gathering

A survey was conducted on the housing equipment management and habits, which was complemented by a telematic interview with home inhabitants. The survey asked about their home profiles (number and schedules of inhabitants), the heating, air conditioning and hot water systems provision and use, main appliances, as well as the use schedules before and during lockdown (as shown in Table 2).

The interviews were focused to study both the way the apartment was used and the user’s personal perceptions of the habits. The inhabitants were asked regarding their habits before and during confinement including alterations or perceived changes:

a) Household cleaning: cleaning habits, use of cleaners, disinfectants, product types changes, as well as the use of air fresheners.
b) Voluntary ventilation (manual window operation): how they performed ventilation, window opening times and frequency.
c) Laundry: estimated washing frequency, hanging and ironing location and type.
d) Confinement management: home entrances and exits, the frequency and number of people and special behaviors during confinement.
e) COVID hygienic special actions: entry/exit cycles procedure, disinfection processes, treatment of purchased products, etc.
f) Tobacco: smokers in the house, daily consumption, smoking place, and related habits.

## 3. Results

### 3.1. Exterior Ambient Quality

As the general purpose of this study was the longitudinal analysis before/after the homes under study due to the need to keep its occupants in them 24/7 and the effects that this process produced on the interior atmosphere. The analysis of the current situation was conducted to gather a general knowledge of the boundary conditions of the homes beforehand and during confinement. While a specific analysis of the evolution of the city atmospheric quality in this period requires a specific and detailed analysis work, which is out of the scope of this work, it is of interest to have a general overview of the outdoor situation in both the prior period and during the main period of confinement.

In general, the emergence of COVID-19 significantly influenced the emission of atmospheric pollutants, especially those linked to road traffic. According to information from the Madrid City Council, the closure of schools caused a drop in traffic of around 8.5%. The subsequent confinement, in which work activity was allowed, produced a significant decrease in traffic intensity, which was between 66% and 70% of a working day equivalent. This was somewhat more noticeable on the weekends, where it reached a reduction of up to 78% reduction. The period of limiting activity to the strictly essential, however, had less of an impact, with an additional drop of only 6% in vehicle movement over the previous week [55].

The external environmental situation of the urban environment significantly improved in all indices, mainly due to the drastic reduction in road traffic and public transport. However, the heating systems of the city buildings (mainly RSI: residential and services buildings) remained a significant source of pollution at the urban level—the average second source of pollution of the city with around one-third of the NOx and PM contributions [56].

**Figure 4:**
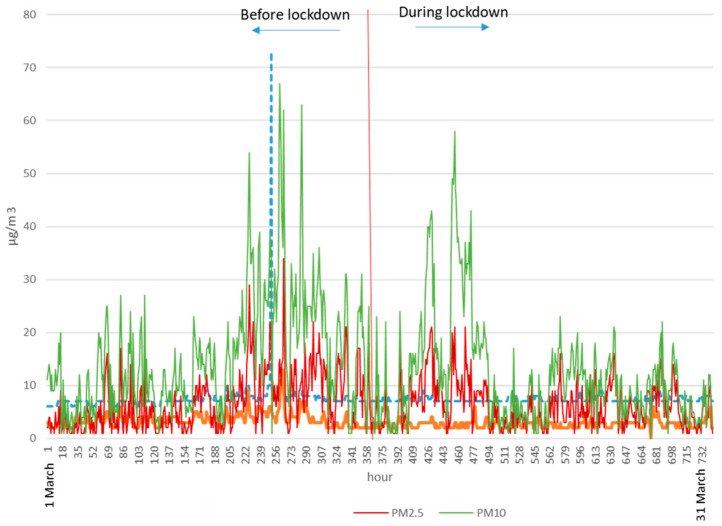
Concentrations of PM_2.5_ and PM10 outside in Madrid before and during home confinement. (Source Sistema Integral de Calidad del Aire de Madrid).

**Figure 5:**
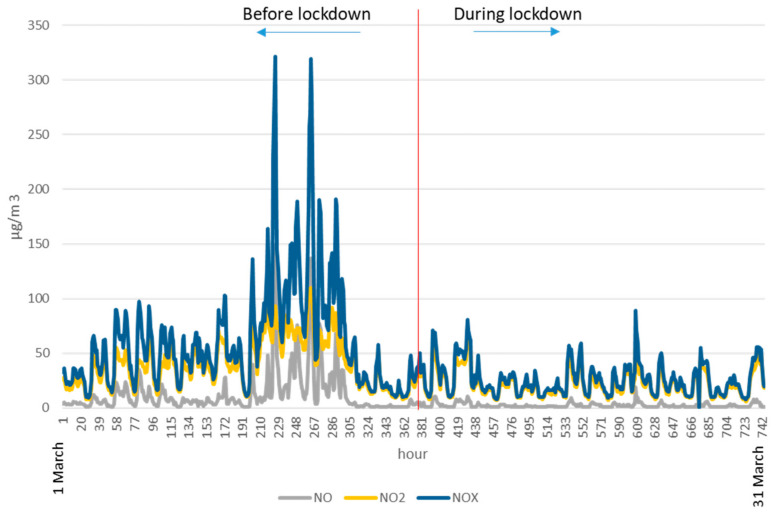
Concentrations of NO, NO_2_, and NOX outside in Madrid before and during confinement. (Source Sistema Integral de Calidad del Aire de Madrid).

Those systems maintained a base level of the most common external pollutants, in particular in the most intense phase of confinement at the end of March and beginning of April when the outdoor temperatures were the lowest of the period, which was fundamentally reflected in the NOx and PM as can be seen in Figure 4 and Figure 5, although with an oscillating profile closely linked to the weather conditions.

Figure 4 shows the concentrations of PM_2,5_ and PM_10_, outside in Madrid before and during home confinement. Figure 5 shows the concentrations of NO, NO_2_, and NOx, outside in Madrid before and during home confinement.

For a general characterization of the city’s atmosphere, we can select, as the main indicators, the PM and NO_2_ values as key parameters of the Air Quality Index (AQI) National System [57], which provides continuous information to the citizens on environmental quality.

In general, the situation of particulate matter in the period analyzed was acceptable to good for the usual standards. There was an average PM_2.5_ daily mean of 11.04 µg/m^3^ in the pre-confinement situation—an AQ index of good (11–20 µg/m^3^)—with only some days rising up to 24 µg—a regular AQI (21–25 µg/m^3^). This situation evolved to a significant reduction during the main period of confinement, with the average of the daily means set to 7.10 µg/m^3^—an AQI of very good (0–10 µg/m^3^)—with only a few days (8.88%) rising above 10 g/m^3^ (maximum of 13 µg/m^3^), which is a very suitable condition with a reduced presence of fine particulate matter in the city environment. With regard to the value of PM_10_, the influence on the period was even smaller with most days located in very good ranges—an AQI of very good (0–20 µg/m^3^) and only a few days prior to confinement with values up to 32 µg/m^3^.

The overall situation regarding NO_2_ was similar, with an initial stage at acceptable values for the typical city period, with average hourly values usually in the *very good* band (46.73% of the period) or good (51.79%)—the AQ Index ranged from 0–40 and 41 to 100 µg/m^3^ with 1.49% of the hours above 100 µg/m^3^ but not surpassing the 110 µg/m^3^ value, and far from the limit of 200 µg/m^3^ set by European legislation [6] as the health limit value.

During confinement, the situation evolved to very good in a generalized way (AQI 0-40 µg/m^3^) with the 90.93% of the hours within this range and only 9.07% in good condition. The peak value was 64 µg/m^3^. With regard to NOx, while this were not included in the city’s air quality indicator system and there is no regulatory limitation for their values, it is equally of interest to comment on their values during both periods. Prior to confinement, as in the NO_2_ case, their values were typically relatively contained with an average hourly average of 66.46 µg/m^3^ without reaching the alert threshold situation (average hourly concentrations of more than 400 µg/m^3^ maintained for 3 h), with only two point short peaks reaching 320 µg/m^3^.

In the confinement phase, these values became quite small with an average hourly average of 25.77 µg/m^3^ and a maximum peak of 89 µg/m^3,^ far from the alert thresholds. Although the indoor direct effects of these species are not included in this work, an analysis was performed to verify that no special or singular situations occurred in the outdoor atmosphere that could have generated anomalies in the previous periods or during confinement. The average values tended to decrease as the month of April progressed, and the outdoor temperatures rose, which, with the traffic contained by the restrictions, is linked with a reduction of the heating uses.

As complementary information, the tropospheric urban ozone levels were low to very low, for both periods, mostly due to the climatological conditions. An average 8-h mean of 41 µg/m^3^ prior to the confinement and 57 µg/m^3^ in the lockdown time were measured, figures that are below the target value for the protection of human health (120 µg/m^3^, eight-hour average) as in DIRECTIVE 2008/50/EC [58]. The atmosphere situation with regard to the ozone concentration allowed for the very good or good classification (0–80/81–121 µg/m^3^ hourly mean) using the Air Quality Index of the city during both periods, prior and during confinement, with all the hourly means under the 100 µg/m^3^ figure and below the legal public information threshold (180 µg/m^3^ hourly). Therefore, for practical purposes and in terms of longitudinal analysis (before/after) given these values, we considered that the presence of tropospheric ozone was not very significant in the modification of the interior conditions of homes due to confinement. In the case of having occurred during the summer, we would expect these figures to be higher, given the city’s historical records.

### 3.2. Information from Users

Case study 1 has the only person who was active outside the home during confinement—being considered an essential worker. In this case, while there was an increase in the working hours, they had an irregular profile. Working-age adults in case studies 2, 3, and 4 maintained their activity during confinement through telework, leading to an increase in the use time for computers and printers.

The routines of entering and leaving the home were severely restricted. In general, only one person from each household was the one who habitually left. Except for case 1 in the work periods, the rest were specific outings of very short duration for the acquisition of basic supplies or pet walks (as established by the regulation of the alarm state). The rest of the inhabitants remained in the house without leaving it during the entire period. The routine for entering and leaving the home is shown in Table 3.

During confinement, all cases reported an increase in the time of use of television and other electronic entertainment equipment (video consoles, tablets, audio systems, etc.). One of the most significant aspects reported was the intensification of household cleaning, also affecting households without regular exits/entrances.

In all cases, the subjects indicated that their intensity and periodicity were increased. The types of products used were generally scaled up, starting from the usual household cleaners with soap and conventional detergents (most of them were aqueous solutions of surfactants). Disinfectants and cleaners of greater power were also introduced. Such new additions are products, including sodium hypochlorite and biocides (mainly PT2 as in BPR No 528/2012 [59], both in liquid agents and aerosol—with/without propellent). Likewise, a widespread use of hydroalcoholic gels (PT1-BPR No. 528/2012) was also reported in all homes. These products were widely used for the disinfection or treatment of not only housing surfaces but clothing, footwear and purchased products, including food packaging.

There was an increase in the use of air fresheners, electric diffusers in case 1 and 2, as well as aerosols in all others. This use was spurred by a feeling of inadequate odors due to the increased periods at home. In case 1, it related both to the presence of pets (animal odors) and to the effects associated with the presence of a regular smoker.

Although there was awareness of the inhabitants regarding a certain alteration in ventilation hours, this did not result in actual modifications to the typical previous routines of each user (opening time, number of windows, etc.) beyond the shift schedules—more by the alterations of other routines than by deliberate decisions. Ventilation was not perceived as a key element either in the importance nor the need for intensification. The main driver in the four cases for routine evolution was the arrival of the spring weather—at the end of the confinement period.

Washing the clothes of people who remain confined suffered a significant decrease in frequency and load, compared to the typical values [59]. On the contrary, the frequency of washing increased with respect to people who leave the home, either to work (CS 1) or to those who did so for the acquisition of supplies. The place for drying clothes and ironing remained the same in all homes before and during confinement. Regarding drying, it was in all cases natural, without the use of a tumble dryer, a very general practice nationwide in Spain [59]. In all cases it was exterior, either in facade hangers or building wells.

During the lockdown, special routines were developed for entering the home: in all cases shoes and clothing that had been taken outside were removed for disinfection when entering the home. In cases 2 and 3—for supply acquisition—the clothes are washed immediately, like those used during worktime for the CS 1 inhabitant. For CS 3, the clothes were aired daily using an outdoor patio as a sanitation mechanism.

The CS1 worker, with a high exposure activity, reported that the only element that was implemented at work for safety was the use of face mask. There was no type of locker or changing room or a specific place to change work clothes or to sanitize them in the workplace, delegating this activity to the home environment.

The user linked to outside work was also the only habitual smoker in the sample. However, they reported that there was no smoking indoors but through a window to the exterior to avoid the direct entry of smoke in the house. As for tobacco consumption habits during the lockdown, they were reduced inside the home, transferring most of the smoking to their (extended) working hours.

### 3.3. Indoor Environmental Quality

#### 3.3.1. Temperature and Relative Humidity

The month of March 2020 in Madrid was thermally usual, with average temperatures around the typical values (average temperature of 11.4 °C and an anomaly of +0.2 °C), although there were differences between the beginning of the month and the end: the highest temperatures occurred on 11 and 12 March (the days prior to the confinement), even exceeding 25.0 °C at some points, decreasing toward the end of the month. The minimum occurred on 31 March with a value of 0.1 °C, this also being the day of maximum rainfall. The month was, in general, very humid with rainfall above the typical values (57.3 L/m^2^). The month of April, when most of the confinement was developed, and especially the severe restriction phase, began with low temperatures (minimum of 3.4 °C) reaching the highest maximum temperatures around 25 April at 23.5 °C. In general, the month was warm (average temperature of 13.8 °C and +0.9 °C of anomaly). Like the previous month, April was also very humid (the sixth rainiest month of April in the 21st century with 16 days of precipitation including storms) [60]. The period prior to confinement had an average outside temperature difference of +2.5 °C with respect to this the confinement period.

The dwellings presented a certain heterogeneity in their air temperature values (Table 4 and Table 5), with the median temperature values in the period prior to the confinement being between 22.5 and 26 °C—these being more representative than the averages due to the presence of specific extreme values. During confinement, the dwellings become warmer in general, above all due to continuous human presence in the daytime hours, moving these central values to 23.1 to 26. °C, showing a range between +0.2 to +0.6 K.

ANOVA was performed with an F-ratio of 5675.66 (*p*-value < 0.05) to establish the statistically significant difference between the averages of the eight variables. Using the Fisher’s least significant difference (LSD) test, we established that the sample sets had significantly different means with a 5% confidence. Complementary tests were performed for the other statistics parameters and we established the difference for all cases (*p* < 0.05).

**Figure 6:**
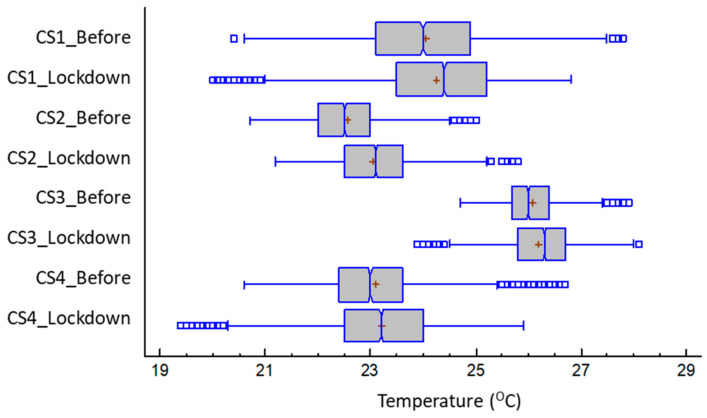
The temperature in the different case studies before and during the lockdown in Madrid.

Typical temperature values were higher than recommended by the national standards (Energy performance of buildings directive EPBD national transposition call for a 17 to 20 °C profile for winter and Heating Ventilation Air Conditioning HVAC regulation for a set-point of 21–23 °C) with most averages being over this threshold for both periods (Figure 6).

These relatively high values standards expected may be associated with the low capacity of the thermal envelope to moderate the thermal wave, since most of the samples were dwellings from the 1950s without thermal insulation and, therefore, have low radiant temperatures during the winter. Therefore, high air temperatures must be used to achieve comfortable conditions, which becomes more evident as the occupation of the dwellings increases. Lower temperatures were usually found in CS 4 despite the building having particularly sensitive inhabitants, such as children and an elderly person.

This contemporary dwelling was built under the requirements of the first transposition of the EPBD and so had a greater insulating capacity of its makeup; therefore, a higher indoor radiant temperature allowing a lower heating air-temperature was set to achieve comfort. The minimum values remained high, around 20 °C, except in CS 3, which was somewhat higher. Based on its quartile distribution (Figure 6), the dwellings were rarely below 21 °C, indicating that heating was continuously used even during periods of ventilation and dwelling cleaning. This aspect, although indicative of a maintenance of comfort, should be considered in terms of energy impact, especially during confinement where the requirements increased.

All the houses kept the heating on in March, although with different patterns. CS1 is the dwelling that had the heating switched for the most hours per day, a total of 12 h. Dwelling CS3 also had the heating switched on continuously for nine hours, and both with fixed power regulation. In contrast, in the other cases, the boiler operates intermittently with schedules programmed in CS4 and CS2 governed by a thermostat (Table 6).

**Figure 7:**
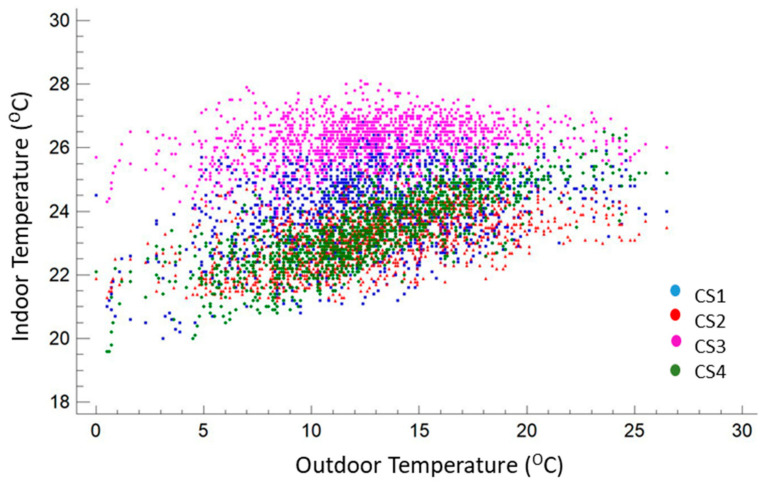
Outside temperature vs. inside temperature in the case studies in Madrid. CS1 (blue), CS2 (red), CS3 (magenta), CS4 (green).

**Figure 8:**
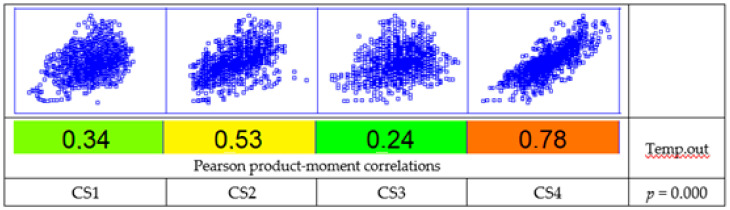
Pearson product-moment correlations in the Madrid case studies.

Although the dwellings had a heating system with a continuous operation (from 24/24 to 9 h a day), their control—when provided (CS 2 and 3)—and/or power sizing did not seem to provide a moderately stable environment, introducing significant oscillations and a thermal evolution linked to the external conditions, as shown in Figure 7. For verification, a correlation analysis was conducted by applying the Pearson product-moment (Figure 8), in this case between the exterior and interior temperature to evaluate the strength of the linear relationship between the variables. The *p*-value was used to verify the statistical significance of the estimated correlations (in this case, correlations significantly different from zero with a confidence level of 95.0% for all cases). CS3 had the weakest relationship, being closest to stable thermal control, with a value of the coefficient of 0.24. Conversely, dwelling CS4 was the most directly influenced by the evolution of the external temperature, with a value of the coefficient of 0.78 in the correlation. This behavior may have been associated with the highest permeability of the group (n_50_:8.2) with a somewhat high value in relation to the typical values [52] with a higher outdoor air infiltration rate.

Because the weather was generally wet during this period, outdoor relative humidity (RH) values were typically high, averaging between 58% and 67% before and during the confinement period, respectively. However, the influence on the interior atmosphere of the different cases was not significant, mainly due to the action of the heating systems. These keep indoor relative humidity in ranges typical for heated spaces, around 40% RH. The greater temporal extension of the presence of occupants did not seem to generate appreciable alterations. The pre- and during-confinement values only showed a slight increase, but without a clear statistical significance when comparing the distribution pairs (*p*-value > 0.05), contrary to the temperatures. This weak effect was also related to the increase in indoor temperatures, which compensates, at least in part, for the foreseeable increase in vapor pressure due to the longer presence of occupants in the dwellings. Notably, the CS2 dwelling usually had high interior humidity (between 55% and 78%), although this situation occurred both before and during confinement, so the operation of the dwelling did not significantly change. This dwelling was the most airtight, with a very low figure for a dwelling of this period (n_50_ 1.2 r^−1^) [61]. In addition, it had one of the lowest available living volumes, around 50 m^3^ per person. Both factors, together with reduced ventilation, fundamentally oriented toward energy conservation, seem to be responsible for these interior humidity values even with the operation of the heating. This aspect for CS2 was also present in the average indoor values of the pollutants, as noted in the following sections.

#### 3.3.2. CO_2_ Concentration

Although CO_2_ is typically considered the main indoor pollutant index, its main role when monitoring indoor environments is as an indicator of the effectiveness of ventilation and the renewal capacity of the indoor atmosphere. In general, work on IAQ adopts CO_2_ concentration as an indicator of actual ventilation rates [62,63,64,65,66] or as an index of indoor air quality [65,67]. Therefore, although it is not considered a major indoor air pollutant, high levels of CO_2_, above 1000 ppm (corresponding to a ventilation rate of approximately 8 L/s per person [68]), often indicate poor ventilation and the presence of other pollutants. Adverse health effects are also associated with levels above 5000 ppm due to oxygen displacement in the air [69], a situation not commonly found in homes. The main emission agents are the occupants of the indoor environments, although the frequent use of open combustion equipment such as gas stoves, boilers, and Domestic Hot Water inside the home, especially in social housing, should be considered.

In general, the dwellings started with an acceptable air renewal situation as the CO_2_ profiles indicate, except for CS2. Typical values are usually below the threshold of 1000 ppm for those found in dwellings of a similar type [42]. Median values ranged from 696.1 (single-occupant) to 844.3 ppm (five-occupant). In dwelling 1, the presence of an usual smoker and pets resulted in an more conscious awareness of ventilation, and frequent window opening, although not necessarily adequate or effective, was reflected in the typical values: median of 783 ppm (σ: 225.95).

In dwelling 2, as identified in the analysis of relative humidity, the recorded values were much higher than in the rest of the sample, indicating a limited renewal of the interior atmosphere. Under normal operating conditions (outside of confinement), the dwelling had values well above the threshold recommended by the World Health Organization (WHO) (Table 7), which were different from the rest of the dwellings in the sample indicating poor ventilation routines, as was noticed in the previous section.

**Figure 9:**
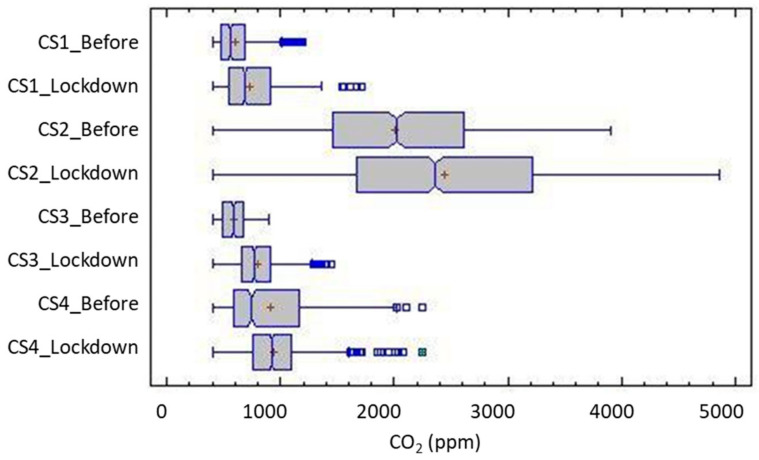
Box and whisker graph of CO_2_ concentration for the case studies before and during the lockdown in Madrid.

The imposition of the stay-at-home order produced a significant modification of the indoor quality of the dwellings, with a general rise of the indoor CO_2_ values in all cases (Figure 9 and Table 8). This alteration affected both the central values, with an increase in the median concentration around 7% to 12%, altering the shape and position of the distribution. Although this increase was foreseeable, given the increase in the time of occupation and permanence in the dwellings, it is especially indicative of a worsening of the indoor air quality under these conditions. Voluntary ventilation appears not to have adapted to these circumstances, either due to specific self-limitations or the absence of awareness and behavioral changes by users.

**Figure 10:**
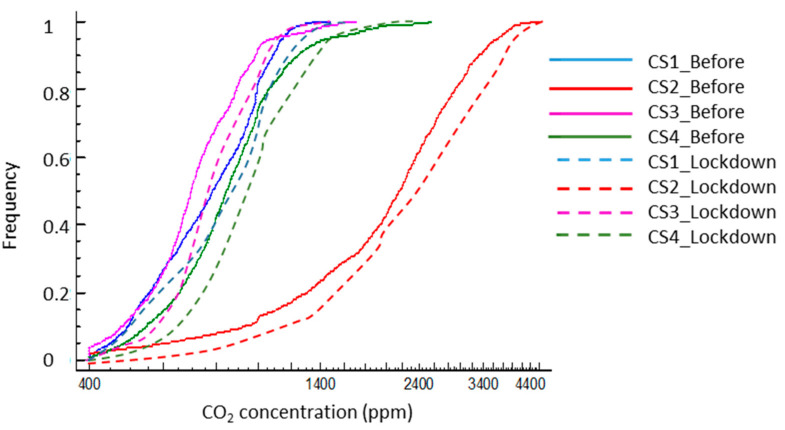
Quantile graph for indoor CO_2_ concentration before and during the lockdown.

Figure 10 shows the quantile graph for indoor CO_2_ concentration. The reduction in density occurred with an adequately ventilated atmosphere in the time analyzed, adopting the threshold of 1000 ppm CO_2_ as an indicator. In general, this alteration was around 10% in three cases, with a smaller increase for CS2, although its values were initially very low, going from a proportion of 0.12 to 0.08 for the threshold of 1000 ppm. This means that during confinement, ventilation in case 2 was only adequate less than 10% of the time. In the rest of the cases, although the values were usually better, the confinement situation worsened the ventilation, with the time of exposure increasing to potentially inadequate atmospheres. Unlike the situation prior to lockdown, during confinement, it can be assumed that the occupation of the dwelling was constant for the whole period, as opposed to cycles of occupation/inoccupation typical of prior to lockdown.

The temporal exposure is relevant, which can be represented by its cumulative distribution (Figure 10). This shows the density of the frequency in which the interior atmosphere could be considered adequately ventilated for each period—assuming 1000 ppm of CO_2_ as the threshold. Confinement decreased the frequency in which the environments were below the threshold in all cases. In general, this alteration was around 10 percentile points in the three cases, with a smaller jump in the CS 2 case, although its frequency values were already initially low (decrease from a proportion of 0.12 to 0.08 for the 1000 ppm threshold). This can be interpreted as that, during confinement, the ventilation in Case 2 was only adequate less than 8% of the hours.

In the rest of the cases, although the values were typically better, the confinement situation worsened the ventilation, with an increase in the time of exposure to poorly suited atmospheres. Unlike the previous situation, during the confinement, we assumed a constant home occupancy, compared to the cycles of occupation/empty-house that are typical of the usual routine.

#### 3.3.3. PM_2.5_

Airborne particles are solid or liquid substances suspended in the air stream, and can include dust, fumes, smoke, microorganisms, mists, and fogs [70]. These particles can vary widely in diameter, with the most harmful in the domestic environment being respirable suspended particles (RSPs), especially the fine inhalable particle fraction sized 2.5 μm or less (PM_2.5_) [71,72,73,74].

The World Health Organization (WHO) has established reference target values both for long-term exposure concentration with annual means under 10 µg/m^3^ and short-term guidance for the 24 h means recommended to be under 25 µg/m^3^ at the 99th percentile (3 days/year) [75]. The European limit value for the annual average is higher at a 25 µg/m^3^ limit. The outdoor concentration of PM_2.5_ for Madrid did not present significant variations between the initial period and the lockdown except for specific peaks, with a daily average around 6 μg/m^3^, although some decrease was observed during the reinforced lockdown (1st extension period).

Therefore, the external situation can be understood as relatively steady. These figures are also typical for the city as it is one of the few European cities that consistently stays below the EU and WHO recommendations [76]. However, there is a general consensus on the need to keep the levels of exposure to PM_2.5_ as low as possible, as it has not been possible to establish a minimum safety threshold without health effects, especially inside buildings [77,78,79]. The reduction of exposure has especially positive effects on sensitive inhabitants, such as individuals with respiratory conditions, the elderly or children [80,81]. Likewise, there appears to be evidence that exposure to PM of internal origin is more aggressive in the respiratory effects than of external origin, with a greater capacity for lung response [82,83]. As a reference, the typical mean residential indoor concentrations in Europe range from 7 (Helsinki) to 21 μg/m^3^ (Athens) [84] and up to as high as those in Italy with 50 μg/m^3^ by Gotschi et al. [85].

Although there are innumerable outdoor sources, there are also internal sources responsible for the emission of particles, which can assume a special role in situations of high intensity use of indoor spaces, such as during a confinement situation. One of the main sources of particles inside residential buildings is cooking, as food preparation causes the emission of vapor, smokes, and aerosols [86,87] and smoke and effluents can be released indoor if there is a lack of a suitable extraction system and by indirect paths due to the possibility of gas-reentry through the infiltration airflow [88,89]. While not as common in residential buildings as they are in offices and public buildings, the presence of printers may also contribute to particle emissions—they are a significant VOC source [90]; however, due to the wide spread of teleworking and tele-teaching during confinement, their presence increased significantly in homes, as well as their use, so they must be taken into account in the analyses.

Another large contributor, which is increasing in homes today, is the use of cleaning agents [91,92], as well as cosmetic and personal hygiene items (deodorants, hair sprays, etc.) [93]. Although these are not typically taken into account, the use of electric air fresheners also has a high capacity for emitting particles [94,95,96]. Since airborne particles are deposited on adjacent surfaces, one of the aspects with the greatest influence on the increase in indoor particle concentrations is the re-suspension due to the movement of the occupants. [88]. This happens very frequent and intensely in indoor environments with regular movement and a low ratio between volume and inhabitants, such as is the case in homes [97,98]. In addition to the emissions caused by the cleaning products, the cleaning process itself, with surface cleaning, sweeping, and other motions, has been identified as an activity responsible for the resuspension of the particles and, therefore, of increased concentrations in the air [99].

**Figure 11:**
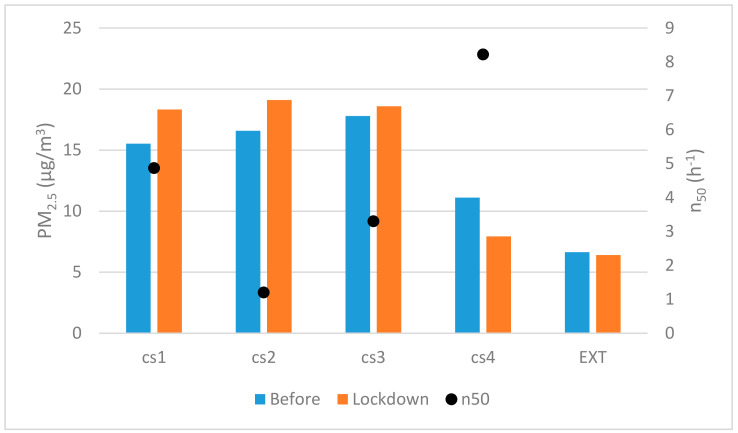
The average PM_2.5_ concentration and airflow leakage at 50 Pa (n_50_) for the case studies for the period before and during the lockdown in Madrid.

In general, the indoor averages of both periods were higher than the city reference outdoor values, as shown in (Figure 11). In the period prior to confinement, the dwellings showed an average between the double (CS4) and triple (CS3) of those found outdoors. During confinement, the averages increased, except in CS4, with central values more than three times the external ones. This issue highlights the significant contribution of indoor sources of emissions, a situation that intensified during confinement.

The higher or lower air-permeability of the envelope did not appear to play a significant role in the indoor PM_2.5_ concentrations through the fabric particle penetration process, as there were high levels of concentration both in airtight homes (CS2) as in average permeability homes (CS 1). Nevertheless, the less airtight dwelling (CS4)—beyond the stock average value [61]—was the one that typically had the lowest PM_2.5_ concentrations. In this case, the airtight homes could benefit from greater air-dilution through the base infiltration, which would contribute to maintaining greater control over the indoor concentration when there are scenarios of limited indoor emissions unlike in CS1. These questions, although require a more comprehensive and in-depth study, which shows a line of interest for future work, and does seem to indicate the prevalence of internal source dynamics over external source dynamics in this situation.

Adequate information on the evolution of particulate matter can be obtained through daily averages, representative of short-term exposure. The representative values before and during confinement are shown in Table 9 and Table 10, represented by the medians and their associated statistics. The typical 24-h average values were initially above 10, although some days had values rise to 57.72 µg/m^3^. In general, as was more evident in cases 1 and 2, the inter-days variation was high, alternating days of low emissions with higher ones, a situation linked to the routines of these households—regular and irregular cycles.

During lockdown, the previously identified values rise was identified in detail as a general increase in the daily average values except for CS 4, with an increase of 12% to 20% in the daily intensity. Likewise, the intra-day variability increased, with low and high emission cycles, reaching mean values of 24 h as high as 90.43 for CS 2. This increase can be associated with two basic aspects: the increase in cooking with regard to the previous conventional routine, which set the base values as is repeated in 24-h cycles, as well as by the intensification of domestic cleaning and housekeeping tasks with respect to the normal situation, which was not developed daily but on separate days. Specific short-time high-peak emissions events were identified in all the homes, although they were not representative (values went up to 1000 µg/m^3^ in certain cases).

Although the period-means (prior/lockdown time) are referred to as a median-term exposure (around 45 days for each set of measures) those figures are above the annual recommended target for WHO. While a direct comparison is not possible, this may indicate a potential risk of exposure if forecasted over time. Of greater interest is the comparison of the WHO limits for the 24-h averages (25 µg/m^3^), which were exceeded for a significant number of days in each period.

In the normal situation, the percentage of days when this threshold was surpassed were: 4.65%, 16.6%, 53.3%, and 2.32% (for cases 1 to 4) and during the confinement these figures increased to 16.6%; 22.9%, and 5% for cases 1 to 3, and CS 4 did not exceed the values on any day in the period. As the epidemiological evidence shows the adverse effects of PM are linked to both short-term and long-term exposures [100]. In this case, the apartments started from an initial profile that was not good—in this period, the actual exposure was lower as the occupation time was also shorter—that worsened during confinement, increasing the risk for the occupants of the dwellings, against the short-term exposure levels and related to the inhabitants—for instance, CS1 and CS2.

**Figure 12:**
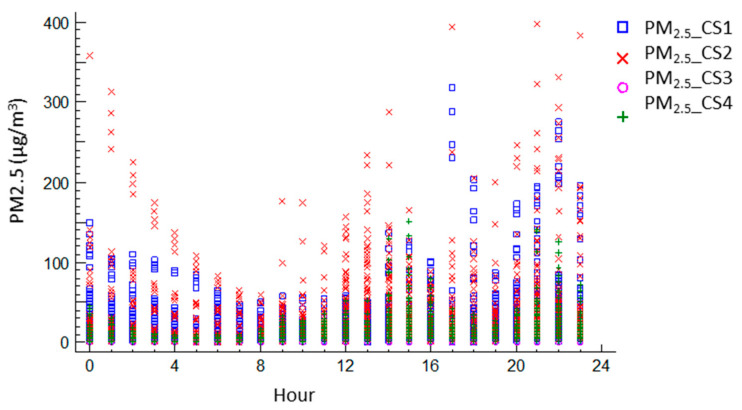
Hourly evolution of the PM_2.5_ of the case studies during the lockdown in Madrid.

As indicated above, in the analysis of the temporal 24-h profile of particles emissions (PM_2.5_) inside the homes, the peaks were associated with the periods of food preparation and daily cleaning, and, as shown in Figure 12, the measures are represented in relation to their temporary presence during the confinement period. Notably, in Southern Europe, the time for dinner is later than in Central Europe, an aspect that seems to have intensified during the confinement period.

#### 3.3.4. TVOC

Along with particles, the most common species in the home environment are volatile carbon compounds (VOCs), represented in this case by TVOCs. Nearly 900 VOCs have been identified in the indoor environment [101], mainly related to interior furniture, finishes, and other construction elements, such as paints, solvents, adhesives, carpets, fabrics, and textiles, but above all to the presence of occupants in the dwelling and their activities, as well as the effect of increased turbulence in the indoor atmosphere caused by movement within the different enclosures (increasing the effect of mixing the pollutants). In urban areas, there are also external sources of VOCs, particularly those related to traffic as well as other combustion equipment. There is evidence that, in European residential and service buildings, the VOC levels are typically higher indoors rather than outdoors—often several times higher [102,103,104]. Likewise, the action of the outside environment, either through ventilation or air permeability, will manifest in a greater or lesser dilution of this component indoors [105].

Exposure to high concentrations of VOCs in air can result in a variety of toxicological effects, including fatigue, headaches, drowsiness, eye irritation, and many others [106,107]. However, the wide variety of potential sources and compositions makes it impractical to measure concentrations of each chemical individually. As such, the concept of total volatile organic compound (TVOC) attempts to address this practical limitation by providing a simple measure of the aggregation of all VOCs without distinguishing between individual chemicals. Although no unified regulation exists for exposure to total VOCs within the home, some general thresholds can be assumed as a result of physiological responses of and disturbances to the occupants. In the range of 120 to 1200 ppb, symptoms of irritation and discomfort may begin to appear in some occupants. Above 12,000 ppb, this discomfort is generalized, with different manifestations depending on the degree of sensitivity of the inhabitants and the species present, with toxicity developing from 10,000 ppb [108]. As reference levels, <120 ppb is considered a healthy environment or VOC-free and >1200 ppb is considered a risk environment with intermediate figures assumed as acceptable but not a risk-free situation. These considerations are general in nature and are highly variable depending on the type of VOC present in the atmosphere [109].

The typical average outdoor levels of TVOC for Madrid (urban center) fluctuated around 14 to 2.85 ppb, although there was a large time variability and weather condition dependency [56]. The most representative species in the city atmosphere were toluene and benzene [14]. During the period prior to confinement, the average daily concentration was Tol: 0.86 and Ben: 0.343 ppb (max. extreme day of 1.75/0.50 ppb). These concentrations shifted to a significant reduction during the period of confinement, going down to a daily average of Tol: 0.146 and Ben: 0.085 ppb (extreme day of 0.39/0.188 ppb) for the last 15 days from March to the end of April. Therefore, there was a significant drop in the VOC outdoor air presence, both on average and extreme days, which may be associated mainly with the drastic reduction of city traffic. This highlights that, during the confinement period, the influence of the outer species, although present in the interior, were not the main drivers of the high interior levels and the evolution observed between periods.

In the case of TVOCs, dwelling 1 was different from the rest as it had one smoker and, even if smoking was not conducted directly in the home, this did present indirect effects that affected the interior quality: the smoke residues on clothing, odors, etc. The high values usually found in this dwelling necessitated conducting the study independently from the rest of the sample. Since relatively high extreme values were found in all dwellings, although only occasionally, the value of the median was more appropriate than the average for representing the behavior of the indoor environment, as the latter is more affected by the presence of extreme values. Excluding CS 1, the medians, both prior to and during confinement, were within the typical findings of similar contemporary dwellings [42], although these were higher than desirable and far from healthy environments. The representative values were within the acceptable label atmospheres, but their high variability, with a variation coefficient of 38.5% to 93.89% for the pre-COVID period and 58.95% to 109.88% for the confinement, indicates periods with high or very high levels of contaminant concentration that became more frequent during confinement.

Table 11 and Table 12 show, respectively, the statistical data on the concentration of TVOCs (ppb) before and during lockdown.

**Figure 13:**
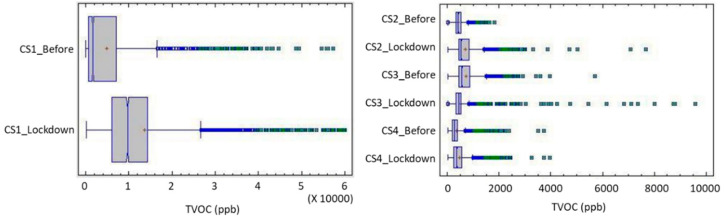
Box and whisker plot of the TVOC concentration for the case studies before and during the COVID-19 lockdown in Madrid.

The frequency over time of the different concentration levels for each time period offers an insight to the kind of exposure that each housing is presenting, which can be expressed through the concentration level temporal percentile. In the normal use of homes (prior to confinement) the exposure of users to high concentrations of VOCs was not very significant—except for case 1—and indoor environments usually maintained an acceptable quality threshold of 1200 ppb (92.5 to 99.5 percentile) (Figure 13), with dwelling 3 being somewhat more exposed. This home was also the one with the highest peak values and width of the distribution—as well as the highest STD value (σ3: 397 ppb).

This can be associated to the profile with a single-inhabitant home and, therefore, the periods of activity/non-activity showed more drastic differences and had a less smoothed profile than others. However, only Case 4 presented significant good quality periods—considered VOC-free when under 120 ppb, with a percentile of 18% for this threshold. In the other situations (1 to 3), periods of low concentration were unusual—with percentile values between 3.0 and 4.3. The periods of occupation were not continuous, and the dwelling had several unoccupied hours.

However, during confinement, the usual concentration increased for dwellings 1, 2, and 4 (increases in the median from 37% to 559%). Excepting Case 1, cases 2 and 4 maintained their central distribution within the acceptable category despite the widening of their standard deviation (σ_2_: 441 and σ_4_: 347), although this threshold did not assure risk-free and the exposure increased as the time spent at home did. The time out of the over 1200 ppb threshold reduced in cases 2 and 4 (88% and 95%, respectively).

In Case 3, the opposite situation occurred, with a reduction in both the mean and the median (–22%), although with a widening of the standard deviation and a particularly high extreme value peak event. This highlights a changes in the indoor routines—less laundry, less use of cosmetic agents, and similar conditions, as described above—and the time below this threshold increased to 97% of the time; therefore, extreme values occurred at very specific moments and for short periods primarily linked to cleaning actions with higher values than before due to more intense actions but with less frequency.

**Figure 14:**
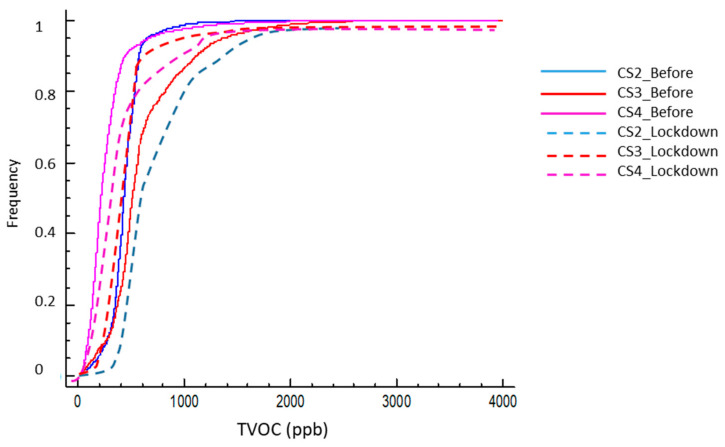
Quantile graph of indoor TVOC concentration before and during the lockdown.

Conversely, in all cases, the almost constant occupation of the dwelling caused the practical non-existence of low periods of VOCs (TVOC < 120 ppb), a situation that can be observed in its probability curves (Figure 14) and the general increase in the values for the 5% percentile.

Dwelling 1, with smoking occupants, presented a unique case. VOC levels were usually high, both prior to and during confinement. Its initial median concentration was 1983 ppb with an average of 5500 ppb due to the presence of extreme values, and with a standard deviation much higher than that found in other dwellings (σ_1_: 7982). This indicated both a large width of the central part of the distribution as well as the presence of occasional situations of extraordinarily high values. This dwelling, under a normal situation, usually had an atmosphere with a high presence of VOC’s, being located most of the time within 354 to 21,696 ppb (5% and 95% percentiles), with very limited periods of good quality atmospheres (<120 ppb, 1.7%; <1200 ppb, 39%). However, the temporary presence of the occupants, with daily work cycles, to some extent reduced the exposure to these species.

During confinement, the concentration of TVOCs in CS1 increased in the medians, extreme values (maximums), and 95% concentrations. The median increased by an order of magnitude of more than five, raising the average above 15,000 ppb. The peak levels surpassed 60,000 ppb. This indicated a very continuous presence of substances at dangerous levels for both short- and long-term exposures. The imposition of confinement worsened the situation, as the hours indoors increased significantly, with the occupants being at risk of almost continuous exposure. In contrast to the previous situation, hours below 120 ppb were negligible (0.025%) and only 0.45% of the period was expected to have TVOC levels below 1200 ppb.

Although smoking related effects were are usually the main factor responsible for indoor peak emissions [110,111] concerning body and clothes residues, as reflected by the periods of activity in the dwelling and the time profile, this caused secondary emissions. To neutralize tobacco odors, in combination with the presence of domestic pets, electric air fresheners were used continuously, generating a continuous emission of VOCs, responsible for the identified high background level as informed by the inhabitants. This situation is compatible with the findings in [21,29,112,113], where the daily clothes laundry of the worker also contributed to the emissions.

The analysis showed a statistically significant difference in the sample distributions between the prior and lockdown situations (at a 95% confidence level) for the contrast tests (T, Mood, and Kolmogórov–Smirnov test K-S test) for each case (*p* > 0.05 in all cases). The data distribution of each case period, especially those for the confined state, better fit the log-log-type distributions compared with the normal.

## 4. Discussion

The sudden change from a presence in the home of between 12 and 15 h per day to practically 24 h/seven days a week (as inhabitants were not allowed to leave the home except for the purchase of essential supplies and work activities for adults), caused an over-strain on the current dwellings, many of them without the adequate capacity to manage this rise in use intensity or lacking the necessary equipment [21,29].

The at-home confinement situation produced evident alterations in the environmental quality of the dwellings. Starting from typical stock conditions for this type of home [42,114], the confinement increased the exposure conditions, in most of the cases, and, when abnormal starting conditions were present (as in CS1), they worsened. Although this situation was expected, the results should arouse concern in most cases, with the identification of multi-factorial risk factors: VOC and PM concentrations, ventilation strategies and effectiveness, continuous inhabitant presence, and a lack of inhabitant awareness of IAQ as well as other aspects not yet identified but linked with ineffective ventilation. This can affect inhabitants both with possible short- and long-term effects. The multifactorial nature of the exposure and effects presents a scenario that should be considered, both for the analysis of the impact on inhabitants and in anticipation of future confinement episodes for the next wave(s) of this/these infection(s).

Notably, together with a significant increase in the sources of internal pollutants, the indoor environmental quality conditions (IEQ) were far from desirable figures. Although the dwellings generally had an acceptable air renewal under the usual standards (combination of infiltration and voluntary ventilation), normal CO_2_ concentrations were below the WHO recommendation threshold, with some exceptions. This does not seem sufficient to ensure the dilution of part of the indoor pollutants (mainly TVOCs and PM) to healthy levels, especially when these are emitted in massive and high frequency amounts.

The concentration of TVOCs in homes over time can be used as an indicator of the level of risk of their environmental exposure. Over time, the alteration produced by the different stages of confinement can be clearly identified. In all cases, we observed an evident change in the profile, although with a certain time lag depending on the specific characteristics of each family unit, reflecting the different situations that may have been experienced in the process.

**Figure 15:**
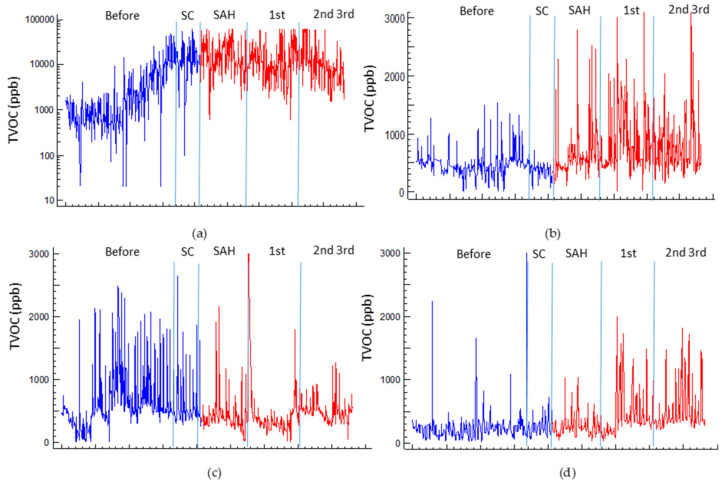
TVOC concentration temporal profiles (ppb) for previous operation (blue) and pandemic declaration-time (red) for Cases (**a**) 1 (*y*-axis logarithmic), (**b**) 2, (**c**) 3, and (**d**) 4.

Case 1 (Figure 15) was different, not only due to its very high emission values, as discussed above, but also due to its own chronological development demonstrating the process experienced by many households in the city. In the period prior to confinement (February), a level around 800–900 ppb TVOCs with cyclical oscillations associated with use was observed. However, the deterioration of the health situation prior to the declaration of the pandemic progressively led to an increased presence in the home starting the weekend of 22 February. At this time, emissions started to trend upward due to the increase in the intensity of use of the home, as well as a possible increase in cleaning tasks. A milestone was noted on 9 March, when school activities were suspended and a preventive confinement—at least partial for the family unit—created the situation that occurred during the entire state of alarm. In this situation, the typical values of emissions were above 10,000 ppb with very wide variations in concentration depending on the emission and ventilation cycles.

In this particular case (CS1), the concentration fluctuations during the confinement peak may have been associated with the intensification of the working activity of a member of the family unit included in the group of “essential workers” who worked during different times during the confinement. The presence of a family member who leaves the home periodically necessitated the intensification of cleaning procedures, personnel, goods, and clothing (more frequent washing of clothes), as well as the incorporation of disinfectant agents more frequently than would be expected in a confinement situation. To this, the presence of the smoker inhabitant and the use of air fresheners and odor neutralizers (electric diffusers and aerosols) must be added.

All these factors had a strong impact on a relatively airtight dwelling—thus, with lower background dilution—siding with the fact that it was the home with the smallest volume per occupant, which allowed concentrations to rise more than in other cases with a greater dilution reserve. Although, conversely, the CO_2_ level was inside the acceptable threshold to WHO standards. This aspect can highlight the need to review these figures, as well as the limitation in the isolated use of CO_2_ as an index of environmental quality. In most of the situations, the use of other complementary parameters will be needed. In several situations, the carbon dioxide levels within what has traditionally been considered acceptable may mask the presence of other pollutants and risks. This situation has been previously identified [115,116,117,118,119] and should be subject to future considerations.

**Figure 16:**
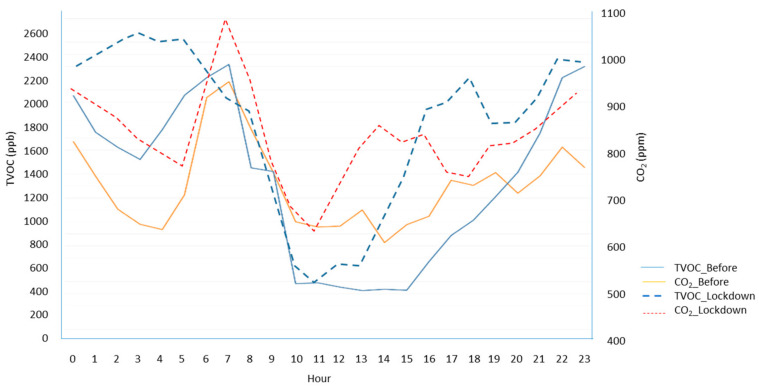
Hourly median profile of TVOC (ppb) and CO_2_ (ppm) for CS1.

It is of interest to observe the hourly profile of the concentrations and the great dependence on the activity cycles of the dwelling, as well as those linked to the voluntary ventilation cycles. Taking case 1 as an example, it is possible to identify the impact on the environmental quality of the activity profiles. In case 1, the emission profile was strongly influenced by the hours of rest, where a continuous base level was maintained, but a slight nocturnal dissipation with actuation of the infiltration, until a morning spike, was linked to the beginning of housing activity, with two valleys, preceding the peaks during the central activity time of the day. The shift in values and the alteration of shape of the distribution is clearly evident in Figure 16.

**Figure 17:**
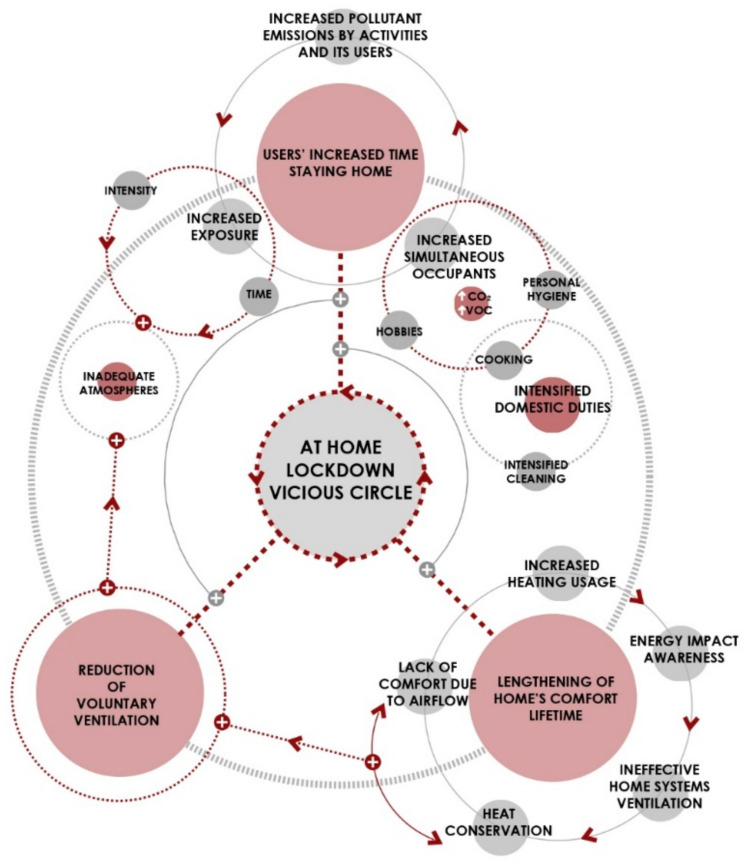
At home lockdown cycle.

Figure 17 shows the process to which the dwellings were subjected during the lockdown.

Future confinement events can be expected related to subsequent waves of SARS-CoV-2—or variants— [120,121,122,123] as well as new pandemic crises [124]. These stay-at-home orders could affect the entire population again or be directed to specific sectors of the population, such as the elderly, immunocompromised, or children [121,125,126], i.e., the more vulnerable parts of the population. The rising in home-schooling can also increase the time spent at-home or in semi-confinement and, therefore, extend the possible risks even without the application of confinement orders. Thus, there is a need for residential IAQ improvement to answer to the new social challenges.

The climatology of the 1st wave period in the city allowed a restrained use of heating, thus contributing to keeping city heating system emissions moderate. Along with the reduction of road traffic, this resulted in a much better urban atmosphere than is typical. As expected, due to climatic conditions the presence of tropospheric ozone (O_3_) in this same period had no meaningful impacts.

While the primary concerns regarding city outdoor pollutants are typically NO_2_/x and particulate matter, and ozone during the warm season [56], city outdoor conditions were somehow acceptable prior to the lockdown and were good when the lockdown was at full force. It, therefore, seems possible to allocate a low influence from outdoor sources in the evolution of the home indoor situation for comparison purposes, with the increase in internal emissions and the ventilation profiles being primarily responsible for the changes.

Fine particulate matter, represented as PM_2.5_, showed evidence of comorbidity in SARS-2 cases, with a relationship between the exposure to the pollutants and the exacerbation of symptoms and increased risk of mortality in the case of contamination of COVID-19 viruses [127,128].

There are risks linked to future confinement episodes in more extreme weather periods. During the coldest climate scenarios—colder than those suffered in this first COVID-19 emergency—the increased use of heating systems, with the associated boiler pollutant emissions, can noticeably increase the presence of pollutants in the city atmosphere. This situation can further aggravate the presence of high atmospheric pressure events during the winter, thus, preventing the atmospheric dilution of contaminants. In future confinements, the residential buildings may have poorer outdoor air to achieve ventilation, resulting in a lower capacity to provide adequate indoor dilution of an often already compromised indoor environment.

Cold periods can also have an impact in low performance buildings and in low-income population groups, which very often coincide. The need for heat conservation and to save energy, particularly in homes affected by energy poverty, can result in an even more significant restriction of voluntary ventilation—the only system available to these houses.

## 5. Conclusions

The need for inhabitants to use heating temperatures above those usually recommended by standards was identified during this period. This corresponded to the need to compensate for low radiant temperatures due to the limited performance of their thermal building envelopes. In most cases, the thermal systems of the homes were unable to maintain stable indoor thermal conditions, with a high dependence on outdoor conditions, which indicates either inadequate sizing/efficiency or underperformance of the building materials to control heat loses (this is of particular note in legacy buildings). This usually results in a limitation on the voluntary ventilation. This situation can be further aggravated in future confinement scenarios (SARS-CoV-2/x next waves) during colder periods—mid winter cold waves—where situations of reduced ventilation to conserve heat may coincide with unsuitable outdoor air quality scenarios, even under situations of a radical reduction in road traffic.

The increase in the intensity of housing use generates high to very high pollutant rising on indoor emissions, which cannot always be counteracted by ventilation in the dwellings, either due to its reduced effectiveness or due to voluntary limitations.

The increase in the domestic use of cleaning and disinfection products during the COVID-19 crisis also have contributed to the increase in VOCs and other chemicals in the indoor environment, just as the generalized imposition of teleworking may have increased the sources of emissions (increase in units and time of use of computer equipment, printers, handling of printed documentation, etc.), although this aspect needs further research to quantify its actual impact.

The presence of inhabitants who leave the home, as in CS 1 in the sample, not only increases the risk of biological contamination but also significantly increases emissions from internal sources into the indoor environment due to the need to intensify disinfection and cleaning (personnel, clothing, and belongings). This increases the risk of exposure of the household occupants.

These factors, together with the intensification of the use of the dwelling, the significant lengthening of the periods of stay (almost doubling the effective hours of use) and, therefore, of the maintenance of thermal comfort, triggered certain processes during confinement, as previously described, with consequent impacts on the risks posed to the occupants.

Given this situation, chemical stress must be urgently reduced in housing, which, being appropriate for daily use, is essential during confinement processes. This situation, despite its extraordinary nature, may become recurring over the next few years, not only in health alert situations, but also during episodes of contamination and other extreme situations that may occur in the future. Given the results, the following recommendations are provided:

- Decrease in daily cleaning products from the homes, encouraging products that are less aggressiveness and have lower emissions (avoiding the use of disinfectant sprays and the high use of biocide cleaners whose loading agents have high VOC).
- Promote the development and incorporation of adequate cleaning and disinfection spaces outside the homes, equipped with appropriate control and confinement elements: air depression situation, extraction systems, adequate drainage, etc. These lock spaces could be located in common areas of buildings or in transitory systems in public road spaces according to the different cases. These spaces have been tested on a larger scale in different countries for controlling access to public buildings. Their use also improves the control of home transmission of viruses such as COVID-19, increasing the security of homes.
- Provide workers with essential services or those with high exposure to biological contamination with specific cleaning spaces in their workplaces, including the handling of clothing and garments, to prevent the need to treat all these elements in the home.
- Reducing the burden of washing clothes and disinfecting belongings can significantly help with alleviating the household emission scenario, as in CS 1.
- This aspect provides an opportunity for its (chemical stress) incorporation into the architectural plans in future developments, and the different disciplines must consider the need to enhance the health safety of homes, both in new buildings and in their retrofitting.
- Promote the limitation and/or reduction of the use of air fresheners (electric, vaporized, or aerosols), odor neutralizers, and all the processes in the interior atmosphere whose principle is based on the emission of substances. General education should be provided on the need to replace all these processes with the most effective ventilation possible, even considering the thermal effects and temporary reduction of comfort.

The awareness of the risks that tobacco poses in the home during confinement situations must be raised, even if this is achieved by opening windows or similar techniques. The derived effects, such as odors and the presence of smoke, tend to increase emissions from compensatory mechanisms (air fresheners, additional washing, etc.).

Action programs should be promoted for the replacement or improvement of the filtration systems of the air-conditioning and heating systems in homes. These programs should be not only based on energy efficiency but also include direct health improvements: coupled with idoneous and efficient ventilation systems, increasing energy efficiency to neutralize the thermal loads in the outside air, and improving voluntary natural ventilation mechanisms and their effectiveness.

## Figures and Tables

**Table 1:** Chronology situation of the confinement in homes in Spain in 2020.

x	Start Date	Progression	Order
**Schools closed (SC)**	9 March	Madrid-region stopped educational activities	
**Pandemic declaration**	11 March	World Health Organization	
**State of alarm**	15 March	Lockout	Stay-at-home (SAH) order
**1st extension period**	30 March	Total lockdown with stoppage of any activity except essential sectors	Stay-at-home order reinforced
**2nd extension**	10 April	As initial stage	Stay-at-home order
**3rd extension**	26 April	As initial stage + Children walks allowed (1 h/day)	Stay-at-home order
	2 May	Confinement relief-measures for general population	Stay-at-home order
	4 May	Resumption (limited) of commercial activities	Onset of deconfinement
**4th extension**	10 May		Deconfinement phases
**5th extension**	24 May		Deconfinement phases
**6th extension**	7 June		Deconfinement phases
	21 June		End of state of alarm

**Table 2:** Characteristics of the dwellings in the study sample (numbers in brackets represents any changes during lockdown).

Case Study		CS1	CS2	CS3	CS4
Year of construction		1954	1950	1950	2008
Floor area (m^2^)		78.5	74	73	90
Free Height (m)		2.93	2.74	2.8	2.7
No. bedrooms		3	3	1	3
No. bathrooms		2	2	1	2
No. Occupants		3	4	1	5
User profile		Couple with children	Couple with children	1 adult	Couple with children and grandfather
Retrofitting		Yes	Yes	Yes	No
Hours per day in the house-occupant 1	Weekday	12 (12)	20 (24)	12 (24)	12 (24)
	Weekend	16 (24)	15 (24)	15 (24)	19 (24)
Hours per day in the house-occupant 2	Weekday	16 (24)	20 (24)		12 (24)
	Weekend	22 (24)	15 (24)		19 (24)
Hours per day in the house-occupant 3	Weekday	12 (24)	20 (24)		10 (24)
	Weekend	22 (24)	15 (24)		19 (24)
Hours per day in the house-occupant 4	Weekday		14 (24)		10 (24)
	Weekend		15 (24)		19 (24)
Hours per day in the house-occupant 5	Weekday				19 (24)
	Weekend				18 (24)
Heating system		Central-heating	Individual boiler	Central-heating	Individual boiler
		Water radiator	Water radiator	Water radiator	Water radiator
Cooling system		Split XD unit in all house	Split XD unit (bedroom and living room)	no	portable evaporative-cooler
n_50_ (h^−1^): airflow leakage at 50 Pa		4.87	1.2	3.3	8.22
Smokers		yes	no	no	no
Gas stove		no	no	no	no
Domestic Hot Water Boiler _(indoor)_		electric	gas	electric	gas
Urban location		Downtown residential street	Residential street with green areas	Close to a main traffic route and green areas	Residential street

**Table 3:** Confinement home entry/exit routines.

Activity	CS1	CS2	CS3	CS4
Work out-of-the-house	daily (1 person)	no	no	no
Telework	no	2 people	1 person	2 people
Supplies	daily (1 person)	each 3 or 4 days	daily	each 3 or 4 days
Pet walking	3 times/day	-	-	-

**Table 4:** Temperature statistics (°C) before home confinement (lockdown). V.C., variation coefficient.

Case	Average	Median	SD	V.C	Min	Max	Range	Quartile_inf_	Quartile_sup_
CS1	24.05	24.0	1.337	5.5603%	20.4	27.8	7.4	23.1	24.9
CS2	22.55	22.5	0.725	3.2148%	20.7	25.0	4.3	22.0	23.0
CS3	26.06	26.0	0.586	2.2479%	24.7	27.9	3.2	25.7	26.4
CS4	23.11	23.0	1.071	4.6347%	20.6	26.7	6.1	22.4	23.6

**Table 5:** Temperature statistics (°C) during lockdown.

Case	Average	Median	SD	V.C.	Min	Max	Range	Quartile_inf_	Quartile_sup_
CS1	24.24	24.4	1.295	5.3401%	20.0	26.8	6.8	23.5	25.2
CS2	23.05	23.1	0.805	3.4896%	21.2	25.8	4.6	22.5	23.6
CS3	26.20	26.3	0.735	2.8071%	23.9	28.1	4.2	25.8	26.7
CS4	23.20	23.2	1.069	4.6058%	19.4	25.9	6.5	22.5	24.0

**Table 6:** Operating hours of the heating systems of the case studies.

Hours	0	1	2	3	4	5	6	7	8	9	10	11	12	13	14	15	16	17	18	19	20	21	22	23	24
CS1											X	X	X	X	X	X	X	X	X	X	X	X	X		
CS2	No schedule, thermostat control																								
CS3													X	X	X	X	X	X	X	X	X				
CS4							X	X	X			X	X	X				X	X	X	X	X	X	X	

**Table 7:** CO_2_ concentrations (ppm) before lockdown.

Case	Average	Median	SD	Variation Coefficient	Maximum
CS1	787.514	783.2	225.915	28.6871%	1480.5
CS2	2136.86	2159.2	897.478	42.0%	4641.7
CS3	731.063	696.1	215.695	29.5043%	1710.0
CS4	892.304	844.3	321.149	35.9909%	2576.5

**Table 8:** CO_2_ concentration (ppm) during lockdown.

Case	Average	Median	SD	Variation Coefficient	Maximum
CS1	854.191	876.1	256.578	30.0375%	1717.0
CS2	2395.72	2308.5	999.038	41.7009%	5000.0
CS3	798.683	774.2	191.666	23.9978%	1497.5
CS4	960.49	923.75	279.717	29.1224%	2252.2

**Table 9:** The daily PM_2.5_ means (μg/m^3^) before lockdown.

Case	Median	SD	Variation Coefficient	Maximum
CS1	14.60	8.91	57.89%	57.72
CS2	12.84	12.21	72.90%	56.05
CS3	16.07	9.14	59.27%	41.43
CS4	10.63	4.77	42.66%	27.64

**Table 10:** The daily PM_2.5_ means (μg/m^3^) during lockdown.

Case	Median	SD	Variation Coefficient	Maximum
CS1	16.37	9.55	49.97%	60.89
CS2	15.47	14.16	74.10%	90.43
CS3	16.94	8.51	53.14	47.27
CS4	7.19	3.22	40.67%	16.73

**Table 11:** Statistical data on the concentration of TVOCs (ppb) before lockdown.

Case	Average	Median	SD	Variation Coefficient	Maximum	5%	95%
CS1 (*)	5550.11	1983	7982.31	143.82%	60.000	354	21,696
CS2	438.453	429.0	169.153	38.57%	2187.8	179	649
CS3	610.77	510.2	397.087	65.014%	5698.2	146	1.367
CS4	272.524	213.9	255.89	93.89%	3954.1	61.5	661

(*) presence of habitual smokers in the home.

**Table 12:** Statistical data on the concentration of TVOCs (ppb) during lockdown.

Case	Average	Median	SD	Variation Coefficient	Maximum	5%	95%
CS1 (*)	15,285	11,090	12,939.9	84.65%	60,000	2.902	46,383
CS2	748.88	591.95	441.54	58.96%	7665.4	338.5	1583
CS3	471.61	401.4	518.22	109.88%	10,530.5	195	876
CS4	438.781	346.2	347.83	79.27%	3964.1	117	1190

(*) presence of habitual smokers in the home.

## Appendix A. Monitoring System Description

An array of electronic sensor is used to continuous monitoring of surveilled parameters.

**Table A1:** Characteristics and uncertainty of the sensors used for on-site measurements.

Parameter	Sensor	Range	Accuracy
Air Temperature	CMOS	−40 to 125 °C	±0.2 °C
Relative Humidity	CMOS	0–100%	±2%
CO_2_	NDIR	400–10,000 ppm	±75 ppm or 10% of reading
TVOC	Multi-pixel metal oxide gas sensor	0–60,000 ppb	±10% (referenced to a gas with an H2 concentration of 0.5 ppm)
PM2.5	Laser-based light scattering sensor	0–1000 µg/m^3^	±15% or ±15 µg/m^3^

The sensors perform real-time continuous measurement, with the system configured to gather data from all the sensors every 15 min in continuous mode and sent to data-storage.

### Appendix A.1. Data Management

The collected data from sensors are stored locally in a local hub—flash storage with circular recording—and sent to a cloud storage. Data are transmitted with a 128-bit AES encryption to protect data packets traveling between devices and the cloud platform. MQTTS protocol is used for real-time communication and HTTPS is used for transactional communication between loggers and the cloud system and transaction information for the webserver process.

### Appendix A.2. Location of the Sensors

Sensors are installed on the living room as the most representative space of the apartment and higher user intensity and commonality. Due to the size and morphology of the homes the indoor ambient can be assumed as a mostly mixed one. They are located in an inner wall centrally located within the monitored space. Mounting height is 1.30 m within “breathing zone”. Monitors are located far from windows inwards (more than half the width of the space). There is no special concern about vent grilles or air diffuser as the houses lack any mechanical or inducted ventilation system.

### Appendix A.3. Measurement Contrast

Testo 435-2—Multifunction indoor air quality reference meter.
